# Diagnosis of tympanic effusions using hyperspectral imaging to prevent unnecessary paracentesis procedures

**DOI:** 10.1186/s12880-026-02611-w

**Published:** 2026-07-24

**Authors:** Maximilian Gänzle, Sabine Löffler, Denis Gholami Bajestani, Michael Unger, Andreas Dietz, Andreas Melzer, Markus Pirlich, Hannes Köhler

**Affiliations:** 1 Department of Otorhinolaryngology, Head and Neck Surgery, University Hospital of Leipzig, Liebigstrasse 12, D-04103 Leipzig, Germany; 2 Innovation Center Computer Assisted Surgery (ICCAS), Faculty of Medicine, Leipzig University, Leipzig, Germany; 3 Institute of Anatomy, Faculty of Medicine, Leipzig University, Leipzig, Germany; 4 Comprehensive Cancer Center Central Germany (CCCG), Leipzig University Hospital, Leipzig, Germany

**Keywords:** Tympanostomy, Hyperspectral imaging, Otoscopy, Middle ear effusion, Otitis media with effusion, Tympanic membrane

## Abstract

**Background:**

Otitis Media with Effusion (OME) is a prevalent childhood condition, affecting up to 90% of children with 10% progressing to a chronic state. It is commonly treated by tympanostomy tube placement. Diagnosis relies on clinical history, otoscopy, audiometry, and tympanometry, but these methods have a high false-positive rate of 15–28%. This highlights the need for an objective, reliable, and non-invasive diagnostic tool to reduce unnecessary surgeries.

**Methods:**

To evaluate the rate of false-positive OME diagnoses in our tertiary hospital, a retrospective database analysis was conducted. To assess the feasibility of advanced imaging, experimental studies were performed ex vivo on human body donors. Sodium chloride solution was intratympanically injected under endoscopic guidance. Hyperspectral imaging (HSI) datasets were acquired using a customized endoscopic system to quantify tissue water content.

**Results:**

An analysis of hospital records identified a false positive rate of 29% (*n* = 4,564) for OME diagnosis, comparing the number of paracenteses to paracenteses plus tympanostomy tubes. In the ex vivo analysis of HSI, visual evaluation of the Tissue Water Index (TWI) demonstrated sensitivity, specificity, and accuracy of 89%, 78%, and 83%, respectively. The mean TWI of all evaluated tympanic membranes was 50.2 (± 7.1) before injection (control) and increased to 62.8 (± 14.8) after injection.

**Conclusion:**

HSI presents a highly sensitive and user-friendly potential future diagnostic adjunct for diagnosing OME, which has shown its potential in an ex vivo approach. Now it needs in-vivo confirmation of the results as its implementation in clinical routine could contribute to avoiding unnecessary surgical interventions, minimizing patient burden and healthcare costs.

## Background

Otitis Media with Effusion (OME) is a prevalent condition in childhood, with an occurrence of up to 90% and the potential for recurrent episodes. In about 10% of cases, OME progresses to a chronic state, leading to complications such as tympanic membrane atrophy or retraction pockets. This condition typically arises from Eustachian tube dysfunction, which may result in biofilm formation that traps bacteria [1]. The resulting conductive hearing loss ranges between 10 and 40 dB.

Diagnosis is based on clinical history, otoscopy, audiometry, and tympanometry. In English-speaking countries, pneumatic otoscopy is commonly used, whereas it is less emphasized in German-speaking regions. Diagnostic challenges, particularly in infants and young children, arise from the subjective nature of audiometry and variability in tympanometry results, with specificity ranging between 72 and 85% [2–4]. This translates to a false-positive rate of 15–28%, which may be influenced by narrow ear canals, cerumen, or patient non-compliance. Additionally, accurate otoscopic evaluation is often challenging in this population due to anatomical and behavioral factors.

In addition to visual inspection, more advanced optical methods for the diagnosis of OME have also been investigated. Optical coherence tomography (OCT) was used to obtain cross-sectional images of the tympanic membrane. This morphological information was automatically classified as normal or OME using machine learning methods [5]. The first study using spectroscopy in the visible and near-infrared range for OME diagnosis proved its feasibility in 258 patients and reported the significance of the water absorption peak at 970 nm [6]. Since water absorbs light more strongly above 1000 nm, several studies have also investigated the shortwave infrared (SWIR) range from 1000 to 1700 nm for imaging of fluid in the middle ear [7, 8]. Carr et al. measured the absorption spectrum of human middle ear fluid ex vivo and demonstrated that the main absorption peaks are based on water as the major chromophore [8]. An overview of various optical and acoustic methods used in clinical studies for the detection of middle ear infections, along with their performance, can be found in Prasad et al. [9]. More recently, autofluorescence of the tympanic membrane was investigated for OME diagnosis using ultraviolet (UV) LEDs in combination with a smartphone-based otoscope and machine learning [10]. Raman spectroscopy has also been explored for otoscopic applications. However, its clinical use remains impractical due to the complexity of the analytical method and its limited penetration depth [11]. A highly advanced non-invasive imaging technique has also been applied to this question. Several research groups have demonstrated the detection of middle ear effusions using ultrasound. Notably, Seth et al. [12] showed that effusions could be detected through a water-filled ear canal in 94% of cases. However, they emphasized that this approach is not practically feasible in awake children. Chen et al. [13] explored the possibility of estimating middle ear fluid characteristics using transmastoid ultrasound. The study suggests that the method can assess middle ear effusion via mastoid ultrasound, but its accuracy is limited.

No effective conservative treatment exists for OME [14]. Current management options include watchful waiting, often combined with nasal balloon therapy, or surgical intervention. German clinical guidelines regarding OME (Status of 2018) recommend myringotomy and tympanostomy tube placement for children with persistent effusions (> 3 months) and hearing impairment [15]. Adenoidectomy combined with tympanostomy has shown the best outcomes in improving hearing thresholds [16].

Surgical treatment must consider the potential for permanent tympanic membrane alterations following tympanostomy tube placement. Additionally, therapy-resistant otorrhea may occur. To prevent otorrhea, the use of earplugs is recommended [17, 18]. Untreated OME can lead to structural changes of the tympanic membrane, including retraction pockets, ossicular erosion, and cholesteatoma, as well as developmental delays in speech and behavior [19–21]. Histopathological changes to the tympanic membrane may also result in altered elasticity of the tympanic membrane [22]. A practical challenge in clinical practice is the latency between diagnosis and surgery. Given OME’s tendency for spontaneous resolution, patients may present without effusion on the day of surgery, leading to unnecessary procedures.

From both medical and economic perspectives, there is a pressing need for an objective, reliable, and non-invasive diagnostic tool to improve preoperative accuracy. Such a tool should be reproducible, cost-effective, and simple to implement. Its ultimate goal would be to reduce the number of unnecessary myringotomies by ensuring the presence of OME before surgery.

Spectral imaging could meet these requirements. This method combines two standard techniques, spectroscopy and digital imaging, in a single device. The resulting image data contains information from multiple distinct wavelength ranges in each pixel. Depending on the method used, these spectral channels can also cover the non-visible range of light and be used for chemometric analysis of tissue, such as determining hemoglobin content. Therefore, this study aims to demonstrate for the first time the technical feasibility of near-infrared hyperspectral imaging of the tympanic membrane for the detection of intratympanic fluid.

## Methods

In order to assess the need for a further diagnostic tool for the diagnosis of OME, we analysed the rate of paracentesis without insertion of a tympanostomy tube (OPS code 5-200.4) for the diagnoses (according to ICD-10) H65.2, H65.3, H65.4, H65.9, H68.0, H68.1, H69.8, and H69.9. This analysis aimed to determine the false-positive rate in diagnosing OME, as evidenced by cases where paracentesis was performed without subsequent tympanostomy tube insertion. As a further OPS code, the number 5-200.5 (paracentesis with insertion of a tympanostomy tube) was searched for in order to compare with the rate of tympanostomy tube insertions performed. For this analysis, cases in which no tympanostomy tube was ultimately placed were used as a proxy measure for a negative indication. It should be noted that this does not constitute a confirmed diagnostic gold standard, as the clinical decision to forgo tube placement may reflect factors beyond diagnostic findings alone. The reported false-positive rate should therefore be interpreted with caution and as a preliminary estimate pending validation against a rigorous reference standard. The data from our internal hospital database system (SAP) from 2012 to 2024 was analysed. In total, we analysed 4,564 patients with an age range from 0 to 94 years and a median age of 3 years. All indications for tympanostomy are based on clinical history, medical examination, and tympanometry.

The technical anatomical examinations were carried out on six human body donors (Table 1). These body donors were stored at a temperature of 4 °C in the Institute of Anatomy at the Leipzig Faculty of Medicine. All body donors had given their informed consent to donate their bodies for research and teaching purposes before their passing. The experimental protocols were approved by the Ethics Committee of the Medical Faculty of the University of Leipzig (129/21-ck). This study was conducted according to the Declaration of Helsinki.

**Table 1:** Data of the human body donors

Body donor ID	Age in years	BMI	Sex	Conservation	Days from death to measurement	Ears included
01	73	33.8	m	cold storage	3	Right
02	87	28.7	m	cold storage	8	Left + Right
03	85	33.4	m	cold storage	1	Right
04	89	25.6	m	cold storage	3	Right
05	89	15.9	m	cold storage	3	Left + Right
06	66	26.4	f	cold storage	4	Left + Right

**Fig. 1:**
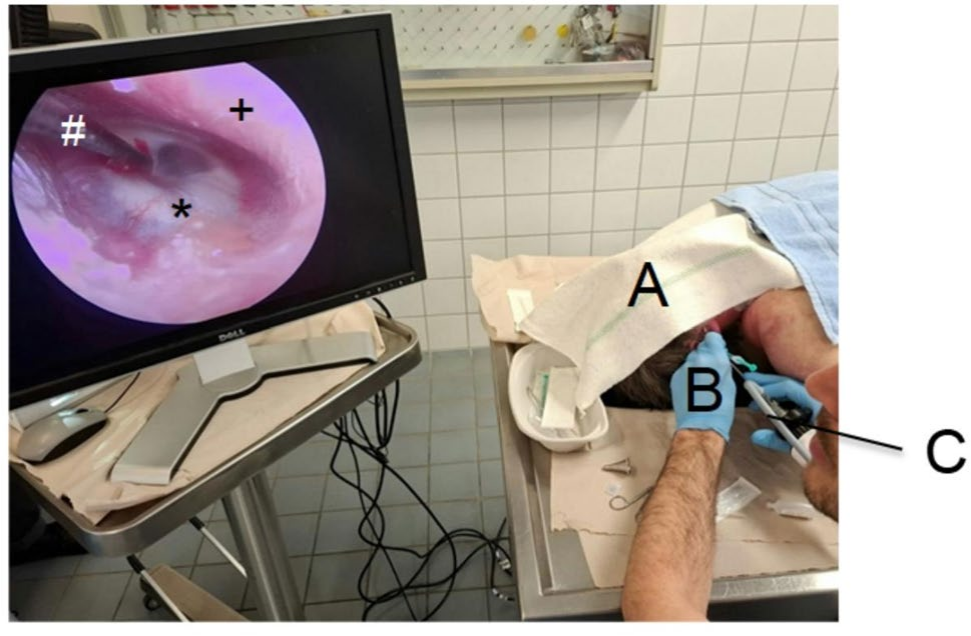
Experimental setup with body donor (**A**), surgeon (**B**), endoscope (**C**) and screen with visualisation of tympanic membrane (*), ear canal (+) and injection cannula (#)

An intratympanic injection of 0.9% sodium chloride solution was performed using a 1 ml tuberculin syringe and an ultra-thin cannula with an oblique cut and a length of 90 mm with an outer diameter of 0.4 mm, 27G (Mediplast AB, Malmö, Sweden) in the sense of an intratympanic fluid injection. This cannula was slightly curved at the base to keep the surgeon’s view clear. The injection was made into the anterior or posterior upper quadrant of the tympanic membrane to prevent the injection fluid from leaking as well as possible (Fig. 1). Beforehand, the ear canal was cleaned if it was blocked with cerumen. The puncture was performed under endoscopic vision. We used a HOPKINS Telescope 0° optic, ICG, 4 mm (28164AC, Karl Storz SE & Co. KG, Tuttlingen, Germany) as endoscope.

Up to 0.5 ml of fluid was gradually injected into the tympanic cavity and a measurement was taken.

### Hyperspectral imaging and data analysis

The tympanic membrane visualization and the acquisition of hyperspectral measurement data were performed with a customized laboratory version of the TIVITA^®^ Mini system (Diaspective Vision GmbH, Am Salzhaff-Pepelow, Germany) [23], which is currently not available for use with 4 mm endoscopes. The device used enables the simultaneous display of a color video and the recording of spatially resolved spectra in the visible and near-infrared range (500 to 995 nm, 5 nm steps). The method used for spectral data recording was push-broom scanning, which required a recording time of seven seconds and resulted in an image size of 720 × 540 pixels. During scanning, the camera should be held as stable as possible to avoid motion artifacts.

An integrated broadband LED light source was used to illuminate the object. The emitted light undergoes specific absorption, scattering, and reflection processes in biological tissue, which lead to spectral changes that can be measured using hyperspectral imaging. The dominant chromophores are oxygenated and deoxygenated hemoglobin. Due to their different light absorption at specific wavelengths, the oxygen saturation of the observed tissue can be determined at each pixel of the image. Although water absorbs light only slightly in the observed spectral range, the local absorption maximum around 970 nm can be used to visualize the spatial distribution of the relative water content of the tissue. A calculation provided by the device manufacturer, as referenced in Holmer et al. [24], was used to quantify tissue water content. The quantification is based on the ratio of reflectance values centered at 960 nm and 885 nm, where the latter is used as a reference wavelength. This derived parameter is referred to as the Tissue Water Index (TWI) with a value range from 0 to 100 and can be visualized using a color map.

The tympanic membrane was annotated in the reconstructed color image derived from the spectral data of each record for the statistical analysis of the TWI after the procedure. Based on this region of interest (ROI), the TWI distributions for each tympanic membrane before (Control) and after fluid injection were investigated. Furthermore, the mean TWI for each ROI was calculated and the distribution over all investigated ears, as well as the Delta TWI (After injection - Control), were analyzed in terms of median and quartiles. Statistical analysis and data visualization were performed using Python (v3.10) with Pandas (v1.5) and Seaborn (v0.11) libraries.

## Results

### Statistical analysis of OME-related operations

**Fig. 2:**
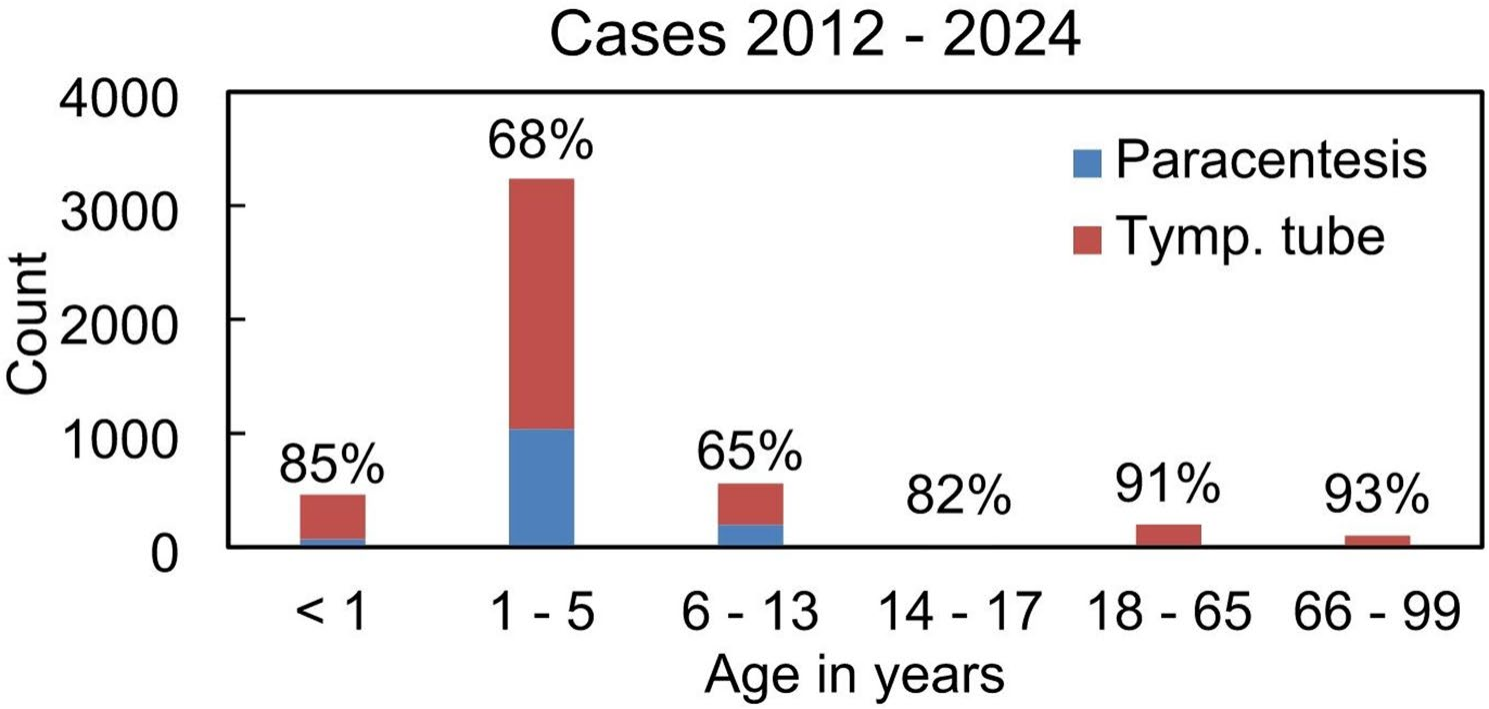
Total number of OME cases (OPS 5-200.4 and 5-200.5) at our department from 2012 to 2024, grouped by patient age. The percentage of tympanic tubes inserted (OPS 5-200.5) is indicated above each bar

From 2012 to 2024 (13 years), a total of 4564 operations (OPS 5-200.4 and OPS 5-200.5) were performed with the ICD-10 codes H65.2, H65.3, H65.4, H65.9, H68.0, H68.1, H69.8 and H69.9. Of these, the vast majority of cases were in the age cohort 1–5 years (Fig. 2). A tympanostomy tube insertion was performed in a total of 3,236 cases (71%) and was omitted in 1,328 cases (29%). This results in a total of 102 paracentesis without tympanostomy tube insertion per year.

**Fig. 3:**
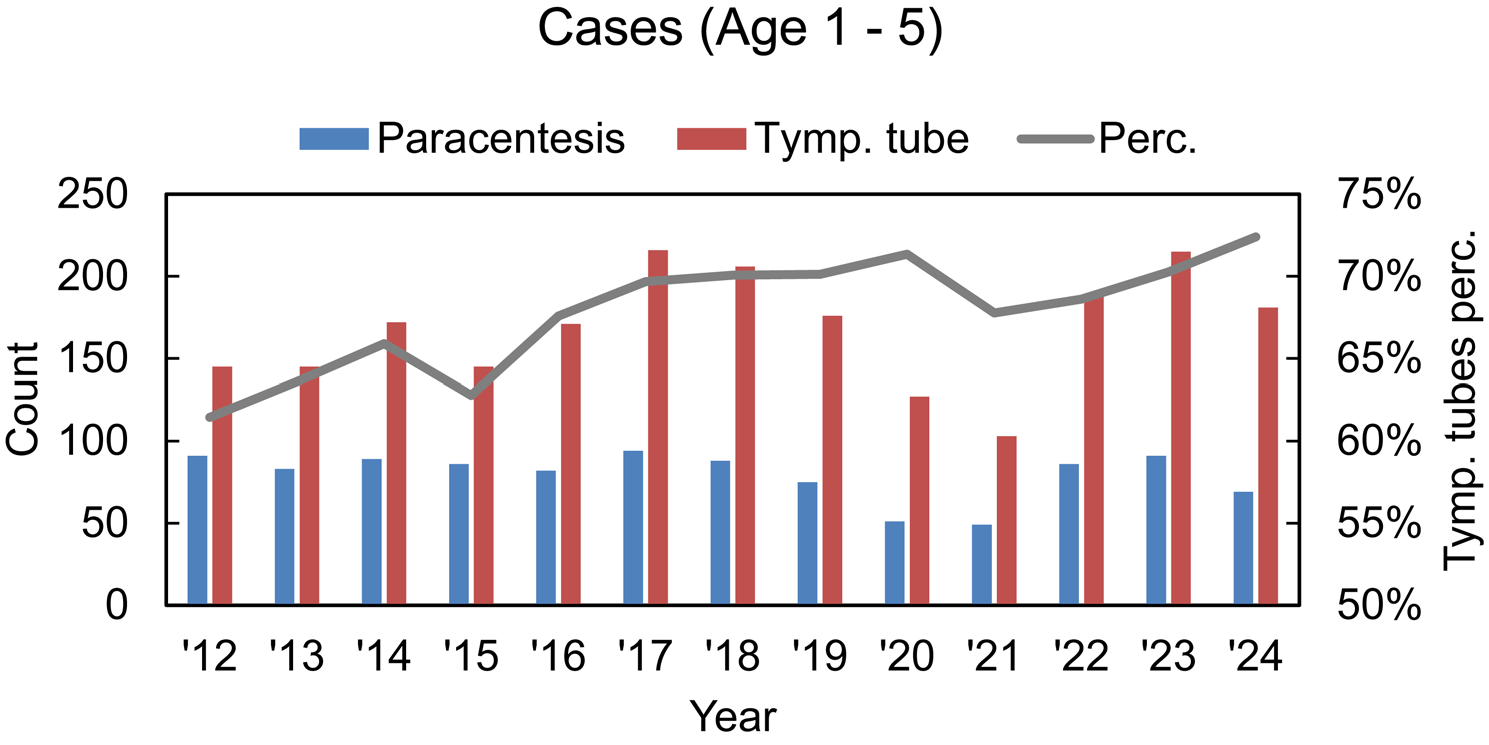
Number of cases with OPS 5-200.4 or 5-200.5 and percentage of tympanic tubes inserted (second y-axis) in the 1–5 years age cohort at our department from 2012 to 2024

In the age cohort 1–5 years, this results in an average annual number of 248 operations, with tympanostomy tube placed in 168 times and omitted in 80 times. An exception is the years 2020 and 2021, in which fewer operations were performed due to COVID-19 pandemic restrictions (Fig. 3). This results in a relatively stable rate of approx. 68% probability for the insertion of a tympanostomy tube in this age cohort, given a clinical indication.

### HSI of tympanic membranes in body donors

Measurements were carried out on a total of six body donors. One tympanic membrane had to be excluded before the measurement due to a rupture (04 L). In two others, water leaked from the injection site (01 L and 03 L). Therefore, three tympanic membranes had to be excluded and nine were evaluated.

**Fig. 4:**
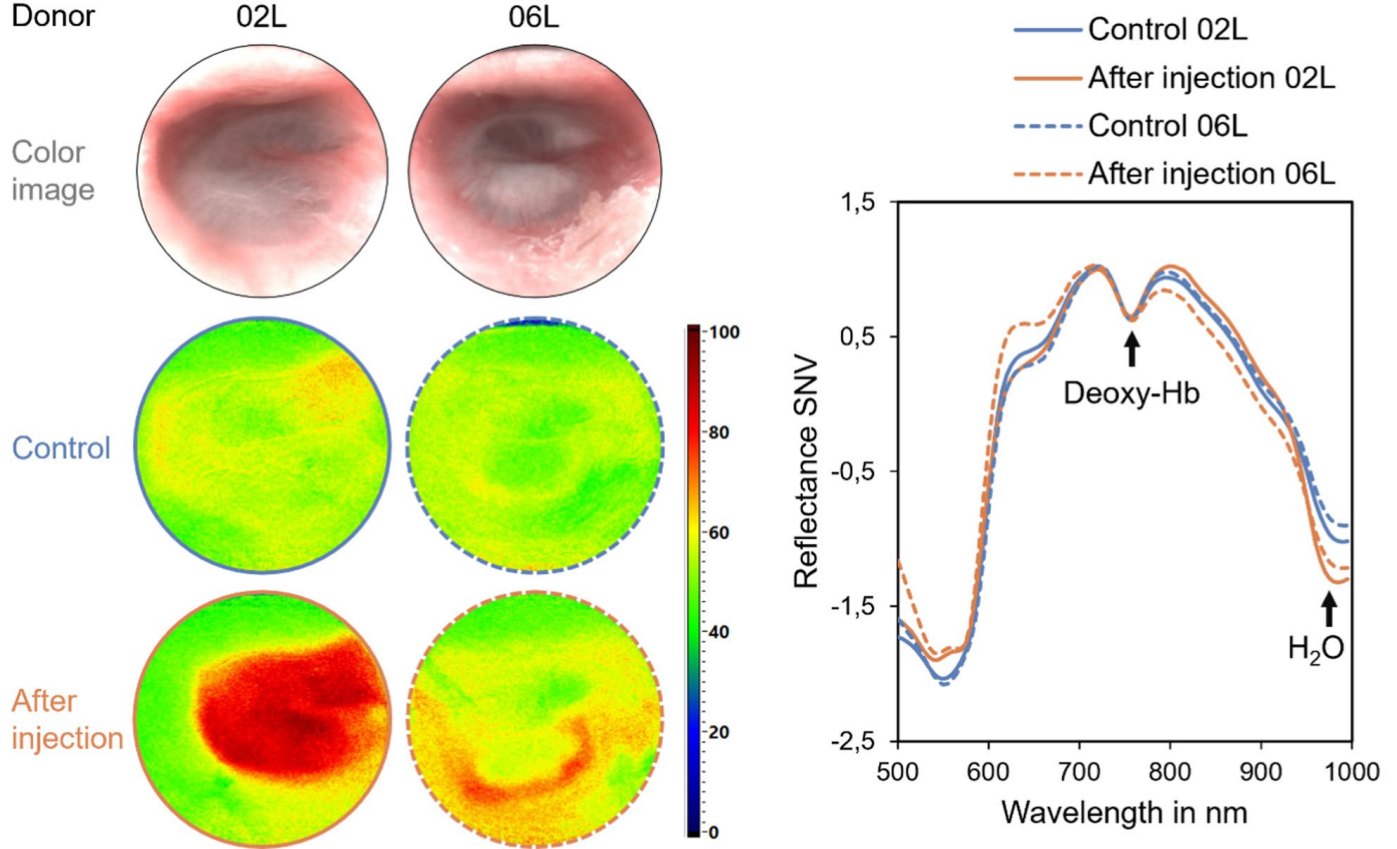
Visualization of intratympanic fluid. Left: Reconstructed color images from the tympanic membrane of body donors 02 (left ear) and 06 (left ear) with color maps representing spatial TWI distribution before (Control) and after injection. Right: Mean normalized reflectance spectra for the two tympanic membranes and both states

The reconstructed color images and TWI maps before and after injection are shown in Fig. 4 for two cases. In addition, this figure illustrates the mean normalized reflectance spectra for these tympanic membranes. The spectral curves clearly show a drop in reflectance at 760 nm, which is caused by the absorption peak of deoxygenated hemoglobin. After the injection, the reflectance spectra show a reduction in the signal at 970 nm, which indicates a higher water content and leads to the increase in the local TWI shown.

**Fig. 5:**
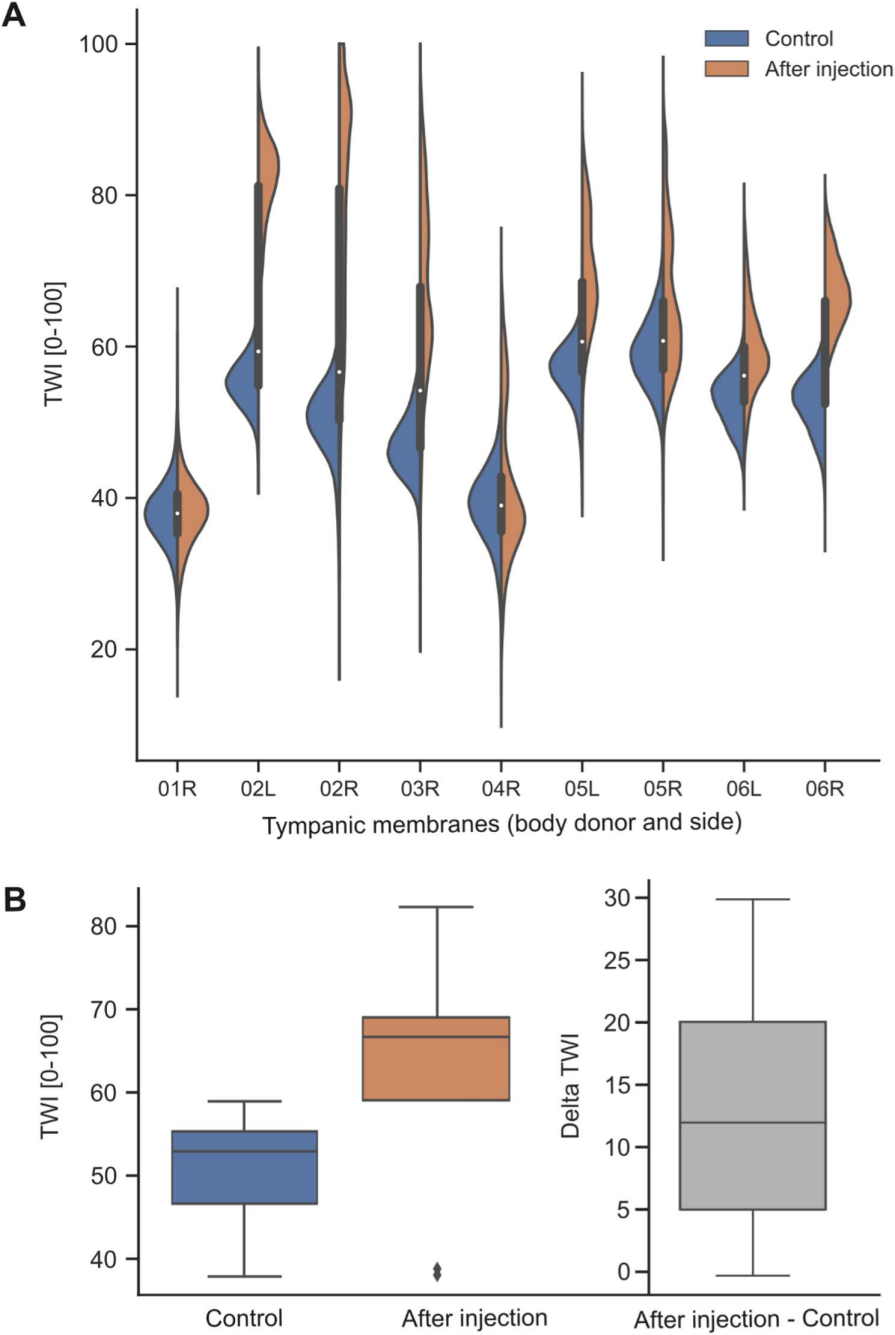
Statistical analysis of all included ears (*n* = 9) and measurements (*n* = 18). Distribution of TWI values (pixel-wise) within each annotated ROI (**A**). Distributions of ROI means and TWI changes (Delta TWI = After injection - Control) per ear (**B**)

A graphical representation of the pixel-wise TWI distributions for each evaluated tympanic membrane can be found in Fig. 5A. In one case (01R), no increase in TWI was observed after injection, while in another case (04R), an increase was seen only in a small area of the tympanic membrane. The visual assessment of TWI spatial distribution by the surgeon was evaluated, as this corresponds to the clinical use case. The resulting metrics of sensitivity, specificity, and accuracy were 89%, 78%, and 83%, respectively. The mean TWI of all evaluated tympanic membranes before injection (Control) was 50.2 (± 7.1) and after injection 62.8 (± 14.8). An average TWI change (Delta TWI = After injection - Control) of 12.6 (± 11.1) was observed (Fig. 5B).

## Discussion

Our analysis of the hospital’s internal database shows that a therapeutic tympanostomy is performed in approximately two out of three cases of OME. On average, 351 surgeries are conducted annually for this indication, with tympanostomy tubes placed in 249 patients, while 102 cases do not undergo this procedure. This results in a 71% likelihood that the indicated therapy is carried out. Consequently, a discrepancy of 29% remains, corresponding to approximately 100 cases per year in a German tertiary care hospital where the indicated therapy is not required. Comparisons with other major hospitals are challenging due to the lack of published data; a PubMed search does not yield comparable studies. In fact, the reported accuracy of diagnosing acute otitis media, a related condition, ranges from 58 to 73% in the literature [6, 25, 26].

The indication for tympanostomy is primarily based on anamnesis, clinical evaluation, tympanometry, and audiometry. However, audiometric assessments in infants and young children often rely on estimations, as brainstem-evoked response audiometry (BERA) is not routinely performed. As mentioned in the introduction, tympanometry shows widely varying sensitivity values, ranging from 72% to 85% [2–4, 27, 28]. This roughly correlates with the findings from our database query on the rate of true-positive middle ear effusions.

A relevant non-invasive diagnostic adjunct would be the use of an HSI camera, as demonstrated in our measurements on body donors in an ex vivo approach, where we achieved a sensitivity of 89%. Since the technical conditions were consistently the same, failures in the tests are more likely to be attributed to inherent test errors. These could be caused by improper injection or drainage of the test fluid (0.9% NaCl), which, unlike middle ear effusion, is not mucous and very viscous but rather fluid (serous). This fluid may have been drained through the patulous Eustachian tube - due to post-mortem tissue relaxation in cadaveric donors - before measurement, or may have pooled in the epitympanum.

Digital otoscopy has gained in popularity over the past few decades, but it does not appear to offer any significant diagnostic advantage over traditional otoscopy in identifying effusions [29, 30]. This limitation is likely due to the constraints of visible light illumination and the inherent variability in human interpretation of the captured images. Preliminary research on this issue has already been conducted. For example, Schmilovitch’s group utilized an otoscope integrated with a spectrometer in the same wavelength range as this work, achieving high sensitivity and specificity in diagnosing otitis media. However, it is important to note that the diagnostic accuracy in this case was based on the subjective judgment of the examining physician as the gold standard, with no paracentesis performed to verify the diagnosis, which limits the reliability of the findings [6].

Monroy’s group explored the use of optical coherence tomography (OCT) to diagnose both acute and chronic otitis media, focusing particularly on evaluating the presence of a biofilm. As in the previous case, the gold standard for diagnosis was the indirect otoscopic assessment, which further weakens the robustness of the results [5]. More recently, Preciado and colleagues developed a prototype OCT otoscope that demonstrated promising results, with a sensitivity of 90.9% and specificity of 90.2%, and the ability to distinguish mucoid from serous effusions [31]. However, as with other otoscopy modalities, a significant challenge remains: variability in user operation and interpretation.

Ultrasound has been extensively studied for detecting middle ear effusion. Filling the ear canal with water to enhance wave transmission has achieved a 94% detection rate [12]. But this approach is impractical in awake children, as water instillation may trigger vestibular symptoms by stimulating the horizontal semicircular canal. Another study investigated the detection of middle ear effusion using transmastoid ultrasound. The method was able to distinguish composition differences of middle ear fluid. However, it is highly operator-dependent, and variations in the scalp or mastoid anatomy may lead to inaccurate results. While promising, this technique requires further investigation [13]. The HSI technique could provide an interesting complement, as it is operator-independent and does not induce vertigo. Although it requires a very calm child as the patient. Combining both methods could potentially improve the detection accuracy.

Two studies have also investigated the use of SWIR otoscopes, achieving high sensitivity rates of up to 90% by applying Random Forest methods to predict OME based on the intensity distribution of the image [7, 8]. Despite these promising results, accurate differentiation between fluid-filled and air-filled middle ear cavities is highly dependent on the light intensity, e.g. due to the measuring distance, and the image output itself provides only limited diagnostic value. It is important to note that the reported SWIR otoscopes do not enable spectral differentiation, but record a single grey-scale image over a broad wavelength range. In contrast, HSI offers a high spectral resolution and thus the capability to calculate ratios between several wavelength ranges. This enables the compensation of external influences and allows distance-independent imaging of the water content.

The primary limitations of this study include the use of NaCl as a solution, which does not match the viscosity of the mucous secretion found in middle ear effusions. This discrepancy may lead to a significantly faster drainage through the auditory tube into the nasopharynx. Another important limitation of this study is the absence of validation in a clinical patient cohort. All measurements were performed under controlled ex vivo conditions using cadaveric specimens, which may not fully reflect the anatomical and physiological variability encountered in living patients. The diagnostic performance reported here should therefore not be directly extrapolated to real-world clinical settings without further prospective validation. Furthermore, the test group was relatively small; however, even within this limited cohort, the measurements yielded strongly positive results early on. Therefore, the trial was terminated after only a few measurements, as continuing with body donors was considered ethically unjustifiable, given the expected redundancy of the findings.

## Conclusions

The current diagnostic approach for OME still has room for improvement. With false-positive rates reaching up to 30%, there is a clear need for more accurate diagnostic tools. HSI offers a highly sensitive and user-friendly quantification of intratympanic fluid and has shown its feasibility and potential in our ex vivo study. These findings represent a proof-of-concept and require validation in a prospective clinical cohort before diagnostic utility in real-world settings can be established. If validated in prospective clinical studies, its use could contribute to avoiding unnecessary surgical interventions, sparing patients from unwarranted procedures while reducing financial strain on the healthcare system.

## Data Availability

The data is available from the corresponding author upon reasonable request.

## Abbreviations

OME: Otitis Media with Effusion
HSI: Hyperspectral Imaging
OCT: Optical Coherence Tomography
SWIR: Shortwave Infrared
TWI: Tissue Water Index
ROI: Region Of Interest
BERA: Brainstem-Evoked Response Audiometry
